# Implantable loop recorders in Brugada syndrome

**DOI:** 10.1093/europace/euac149

**Published:** 2022-09-03

**Authors:** Chiara Scrocco, Elijah R Behr

**Affiliations:** Cardiovascular Clinical Academic Group - St. George’s, University of London and St. George’s University Hospitals NHS Foundation Trust, London, UK; Cardiovascular Clinical Academic Group - St. George’s, University of London and St. George’s University Hospitals NHS Foundation Trust, London, UK

This Letter to the Editor refers to: ‘The evidence for the implantable loop recorder in patients with inherited arrhythmia syndromes: a review of the literature’ by Balfe *et al*. https://doi.org/10.1093/europace/euab256.

**A response to this letter is available: ‘Implantable loop recorders in Brugada syndrome: Authors’ reply’ [https://doi.org/10.1093/europace/euac150] by Balfe *et al*.**

In this issue of the *Journal*, Balfe *et al*.^1^ presented a review of the available evidence on the use of subcutaneous implantable loop recorders (ILRs) in patients with inherited arrhythmia syndromes. The authors must be commended for this effort, which highlights merits and limitations of previous publications on this subject. Unfortunately, our recently published manuscript on the largest experience with the use of ILRs in patients with Brugada Syndrome (BrS) reported to date^2^ seems not to have been included in this otherwise comprehensive review.

We described a cohort of 50 BrS patients with ILRs, mostly symptomatic (two-thirds with previous syncopal or pre-syncopal episodes, almost one-half with palpitations), with a median follow-up of 28 months (range 1–68). The main findings of our study were that an ILR-guided diagnosis can be made in up to 57% of subjects with recurrent syncope (especially those with previous unexplained or suspected arrhythmic episodes) and 50% of subjects with symptomatic palpitations. Most importantly, our study recognized paroxysmal sinus node or AV conduction dysfunction as important and frequent underlying mechanisms of unexplained and even presumed vasovagal/reflex syncopal episodes in these subjects. In addition, we provided a direct comparison to the demographic and clinical characteristics of BrS subjects implanted with an ICD at our institution and proposed who may benefit most from prolonged monitoring with ILRs.

We agree with the points raised by Balfe *et al*. in their manuscript, although some of the answers could have been found in our report; for example, the outcome of extended follow-up of subjects with BrS implanted with an ILR. We concluded that the use of an ILR ‘can be helpful in guiding the management of low-/intermediate-risk BrS patients and ascertaining the cause of unexplained syncope’. Nonetheless, we agree that in the setting of rare cardiac conditions such as BrS and other primary arrhythmia syndromes, further research, including the use of multi-centre registries and trials is indeed necessary to better understand the role of prolonged monitoring in identifying subjects potentially at risk.
